# Clinical immune-monitoring strategies for predicting infection risk in solid organ transplantation

**DOI:** 10.1038/cti.2014.3

**Published:** 2014-02-28

**Authors:** Mario Fernández-Ruiz, Deepali Kumar, Atul Humar

**Affiliations:** 1 Division of Transplant Infectious Diseases, Multi-Organ Transplant Program, Toronto General Hospital, University Health Network, University of Toronto, Toronto, Ontario, Canada

**Keywords:** cell-mediated immunity, cytomegalovirus, immune-monitoring strategies, infection, prediction, solid organ transplantation

## Abstract

Infectious complications remain a leading cause of morbidity and mortality after solid organ transplantation (SOT), and largely depend on the net state of immunosuppression achieved with current regimens. Cytomegalovirus (CMV) is a major opportunistic viral pathogen in this setting. The application of strategies of immunological monitoring in SOT recipients would allow tailoring of immunosuppression and prophylaxis practices according to the individual's actual risk of infection. Immune monitoring may be pathogen-specific or nonspecific. Nonspecific immune monitoring may rely on either the quantification of peripheral blood biomarkers that reflect the status of a given arm of the immune response (serum immunoglobulins and complement factors, lymphocyte sub-populations, soluble form of CD30), or on the functional assessment of T-cell responsiveness (release of intracellular adenosine triphosphate following a mitogenic stimulus). In addition, various methods are currently available for monitoring pathogen-specific responses, such as CMV-specific T-cell-mediated immune response, based on interferon-γ release assays, intracellular cytokine staining or main histocompatibility complex-tetramer technology. This review summarizes the clinical evidence to date supporting the use of these approaches to the post-transplant immune status, as well as their potential limitations. Intervention studies based on validated strategies for immune monitoring still need to be performed.

Despite continued improvement in the clinical management of solid organ transplant (SOT) recipients, infection continues to be one of the leading causes of morbidity and mortality in this population. Two main variables need to be accounted for when evaluating an individual patient's risk for post-transplant infection: the epidemiological exposure (that is, postoperative colonization by multidrug-resistant pathogens) and the ‘net state of immunosuppression'.^1^ The latter emerges from a complex interaction that encompasses multiple factors, including the type of immunosuppression regimen used, its timing and dosage, the presence of underlying immune defects or viral co-infections, and the evolution of graft function.^2^ Thus far, clinicians caring for SOT recipients have relied almost exclusively on the therapeutic drug monitoring of immunosuppressive agents to explore the status of immunocompetence of their patients.^3^ Nevertheless, such an approach appears limited by its unidimensional nature, which does not take into account the large variety of pharmacokinetic, pharmacodynamic and clinical variables that modulate the phenotypic activity of modern immunosuppression protocols.^4^ Moreover, the evidence supporting the utility of therapeutic drug monitoring of agents other than calcineurin inhibitors and mammalian target of rapamycin inhibitors remains less conclusive.^5, 6^

As a result of ongoing efforts to fulfill this unmet clinical need, we now have an expanding repertoire of immune-monitoring strategies that may stratify the odds for developing infection in a given SOT recipient and, eventually, provide the basis for tailored immunosuppression and prophylaxis strategies. Of note, these approaches largely differ in their theoretical background, type of event predicted, assay methodology, limitations and feasibility in daily practice. This review summarizes the state of the art in this emerging field.

## General rationale for post-transplant immune monitoring

The immune response, either innate or adaptive, is the result of an extremely complex interplay between soluble and membrane-bound signaling mediators and specialized cell populations, eventually leading to the initiation of a number of effector mechanisms.^7^ The different strategies proposed for immunological monitoring ultimately pursue to reduce the complexity of such a process—or at least a part of it—into an individual or group of parameter(s) or biomarker(s) that, by means of regular measurements, may provide a dynamic insight into the net state of immunosuppression of the subject and the subsequent correlation with the risk for post-transplant infection. Ideally, the assay on which this monitoring is based should be reliable, sensitive and specific enough, highly reproducible, and its results should be available for the clinician within a short turnaround period to allow timely modifications in immunosuppression or prophylaxis.^8^ The implementation of these strategies must eventually be linked to a feasible intervention that has been proven to provide some clinical benefit.

From a clinical perspective, the approaches to the immune monitoring in SOT recipients could be grouped according to its target into *non-pathogen-specific* or *pathogen-specific*. The first category encompasses those strategies aimed at evaluating the functionality of a given arm of the immune system by means of assays (or biological parameters) with no antigen specificity. Therefore, in most of the studies the predicted event is the occurrence of overall infection—with no further classification according to the clinical syndrome or the causal pathogen—or, at the most, a generic type of infection (that is, bacterial or fungal infection). The nature of the biomarker used may be merely quantitative—such as the concentration of serum immunoglobulins—or provide some functional assessment—such as the intra-lymphocytic release of adenosine triphosphate (ATP) under a nonspecific mitogen. In contrast, the pathogen-specific immune-monitoring strategies rely on antigen-specific assays that estimate the magnitude and functionality of adaptive immune responses generated by T cells or B cells against a given pathogen, usually by measuring the production of Th_1_ effector cytokines (that is, interferon (IFN)-γ or tumor necrosis factor-α) upon stimulation with a known antigen. Although progress has been made in the assessment of different virus-specific cell-mediated immune responses, including Epstein–Barr virus (EBV)^9^ or BK polyomavirus (BKPyV),^10^ we will mainly focus on current developments in cytomegalovirus (CMV)-specific T-cell monitoring, in view of its relative state of maturity and wide potential implications for the management of the SOT population.

## Non-pathogen-specific monitoring

As summarized in Table 1, the proposed strategies for nonspecific immune monitoring after transplantation are notably heterogeneous in terms of complexity, capacity for functional assessment and technical requirements.

### Serum immunoglobulin levels

For some years, the occurrence of secondary *de novo* hypogammaglobulinemia (HGG) was a somewhat neglected immunosuppression-related complication in SOT recipients. Nevertheless, a recent meta-analysis reported that mild (serum immunoglobulin G (IgG) levels 400–700 mg dl^−1^) and severe (IgG <400 mg dl^−1^) HGG occur in as many as 39% and 15% of patients during the first year post-transplant, respectively.^11^ The incidence and clinical implications of HGG have been assessed in kidney,^12, 13, 14, 15^ liver,^16^ lung,^17, 18, 19^ heart^20^ and intestinal^21^ transplant recipients. The mechanisms leading to post-transplant HGG are not fully clarified and are likely multifactorial, including the decrease in CD4^+^ T-cell numbers and its subsequent impact on B-cell activation.^22^ The use of mycophenolate mofetil has been also shown to increase the incidence of HGG in some studies,^12^ presumably through a direct detrimental effect on B-cell function.^23^ In addition, certain graft-specific risk factors have been identified, such as the presence of bronchiolitis obliterans syndrome for lung transplantation^18, 19^ or the administration of steroid pulses for heart transplantation.^20^

**Figure 1:**
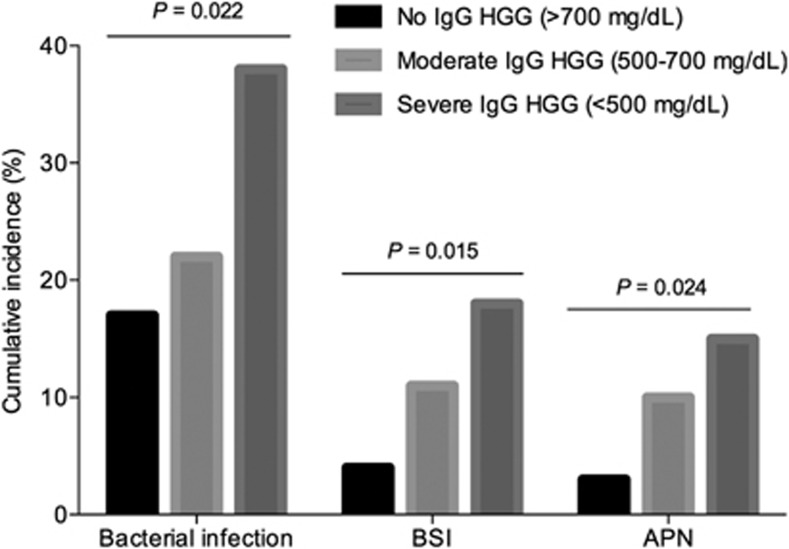
Cumulative incidences at month 6 post-transplant for overall bacterial infection, bloodstream infection (BSI) and acute pyelonephritis (APN) according to the serum IgG levels at month 1 in a prospective cohort of 271 kidney transplant recipients (modified from reference Fernández-Ruiz *et al.*^15^ plus personal data (Fernández-Ruiz M, 2013, unpublished data)).

The humoral arm of the immune response is primarily responsible for the clearance of encapsulated bacteria (that is, *Streptococcus pneumoniae* or *Haemophilus influenzae* type b) by opsonization, antigen neutralization and complement activation.^24^ Post-transplant HGG, specifically of IgG, acts therefore as a good predictor for bacterial infection.^11^ A recent prospective study in kidney transplant recipients found a ‘dose-effect' in the occurrence of infection according to the post-transplant IgG levels, with a clear gradient from mild or moderate to severe HGG (Figure 1).^15^ The impact of IgG HGG on the incidence of other bacterial infections has been also demonstrated for bacteremia^15, 17^ and *Clostridium difficile*-associated diarrhea.^25^ Less intuitively, some authors have suggested that the risk of CMV disease^20, 26^ or invasive fungal infection^17^ is increased in patients with post-transplant HGG. The meta-analysis by Florescu *et al.*^11^ reported that the odds of CMV and fungal infection for severe HGG were 2.89 and 3.69 times higher, respectively, than those for mild HGG. Although the role in the control of CMV replication of neutralizing antibodies targeting the viral glycoprotein B is increasingly recognized,^27, 28^ it could be argued that the detection of low immunoglobulin levels simply represents a surrogate for a higher degree of immunosuppression or poorer clinical status.^29^ In that line, Doron *et al.*^16^ found a lower long-term survival in liver transplant recipients with HGG, even in the absence of a discernible effect on the incidence of infection. Despite this and other limitations (the heterogeneity of studies and the lack of common definitions for the different categories of HGG), this approach to the monitoring of humoral response offers two additional advantages. First, the measurement of serum immunoglobulins by nephelometry is a widely available technique and economically affordable (≈13 US Dollars per determination).^15^ Second, as opposed to other immune defects in transplant recipients, HGG is potentially reversible without increasing the risk of graft rejection, and some studies have already evaluated the preemptive replacement therapy with intravenous immunoglobulin, with promising results.^30, 31^

### Serum complement factors

The complement system constitutes another target for monitoring strategies in view of the relevance of its effector functions (opsonophagocytosis, induction of acute inflammation and cellular lysis) in innate and adaptive humoral immune responses.^32^ The relative contribution of complement is further highlighted in the setting of post-transplant immunosuppression, which is primarily directed against adaptive cellular immunity.^33^ The assessment of complement functionality has classically relied on *in vitro* hemolytic assays (CH_50_ and AP_50_ for the classical and alternative pathways, respectively).^34^ However, the complexity and time-consuming nature of these methods preclude their daily clinical application. The measurement of serum levels of certain components by a more feasible method (nephelometry) represents a convenient proxy for the complement activity. The three activation cascades converge on the third component of the complement to form the C5 convertase (C4bC2aC3b for the classical and lectin pathways and [C3b]_2_Bb for the alternative pathway) and, ultimately, to assemble the membrane attack complex (C5b-C9).^32^ Therefore, this pivotal role played by C3 makes it a good candidate for monitoring. The utility of this strategy has been shown in a prospective study of 270 kidney transplant recipients, in which the presence of C3 hypocomplementemia (serum levels <83.0 mg dl^−1^) at month 1 was as an independent risk factor for the subsequent occurrence of overall and bacterial infection (hazard ratios of 1.9 and 2.1, respectively).^35^ Comparable findings have been reported for liver^36^ and heart transplant recipients.^37^ Unfortunately, the only intervention that seems feasible in a patient with low complement levels consists of decreasing immunosuppression, which in turn could increase the risk of graft rejection.

The functional status of the lectin activation pathway may be specifically explored by assessing the serum concentrations of mannose-binding lectin (MBL), which in turn are largely determined by various polymorphisms occurring in the *mbl2* gene or its promoter region.^38^ Structurally related to the C1q component of the classical pathway, serum MBL can also be easily measured by nephelometry or enzyme-linked immunosorbent assay. In a cohort of 102 liver transplant recipients, the presence of low MBL levels were associated with a higher incidence of clinically significant infection (52% vs 20% *P*-value=0.003). Donor *mbl2* genotype was the strongest determinant of post-transplant circulating MBL levels, a not surprising finding considering that this pattern recognition molecule is produced primarily by the liver.^39^ MBL deficiency has been also linked to the development of sepsis in kidney or pancreas–kidney transplant recipients^14, 40^ or CMV infection after discontinuing valganciclovir prophylaxis.^41^ More studies are needed to determine the optimal cutoff levels and timing for the monitoring of this biomarker. In addition, it remains to be clarified whether the demonstration of MBL-deficient genotypes of *mbl2* or related genes (MBL-associated serine protease or ficolin-2 genes) could avoid the need for post-transplant monitoring of serum levels, as suggested by some authors.^42, 43^

### Peripheral blood lymphocyte sub-populations

**Figure 2:**
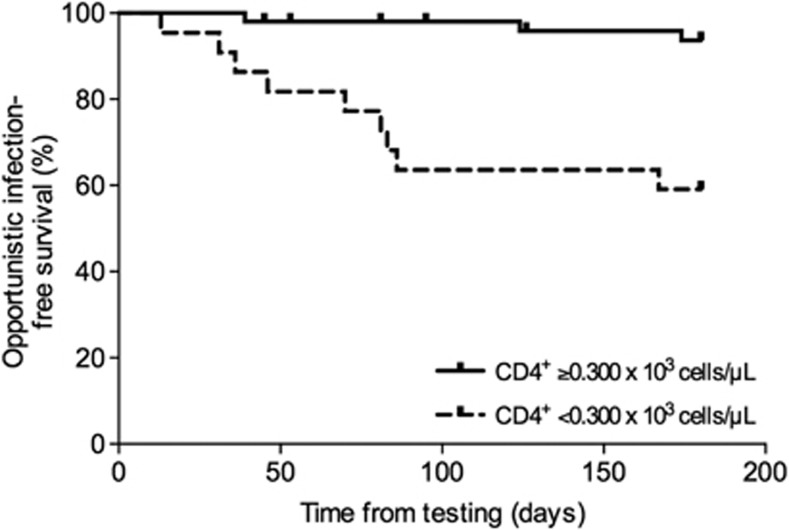
Opportunistic infection-free survival in 82 liver transplant recipients according to the CD4^+^ T-cell count at month 1 post-transplant (*P*-value=0.0001; log-rank test) (Fernández-Ruiz M, 2013, unpublished data).

The administration of lymphocyte-depleting agents (that is, rabbit polyclonal antithymocyte globulin or anti-CD52 (alemtuzumab) monoclonal antibody) for induction therapy or treatment of rejection is a well-established risk factor for the occurrence of post-transplant infection.^44^ Similar to monitoring of patients with human immunodeficiency virus infection, the kinetics of certain peripheral blood lymphocyte sub-populations have been explored as the basis for post-transplant immune monitoring. Calarota *et al.*^45^ regularly assessed the CD4^+^ and CD8^+^ T-cell numbers during the first 8 months after kidney and heart transplantation and reported that those patients who developed opportunistic infections—because of CMV in most of cases—had lower counts as compared with those without. In the specific setting of human immunodeficiency virus patients undergoing kidney transplantation, the presence of a CD4^+^ T-cell count <200 cells μl^−1^ was associated with the occurrence of opportunistic or severe infection.^46^ Various authors have consistently shown that the risk of *Pneumocystis jiroveci* pneumonia after kidney transplantation is increased in recipients with low CD4^+^ T-cell counts,^47, 48, 49^ and it has been suggested that the dynamics of peripheral blood lymphocyte sub-population may help to guide the duration of prophylaxis with trimethoprim-sulfamethoxazole, similarly to human immunodeficiency virus patients.^48^ In a recent prospective study with 82 liver transplant recipients, having a CD4^+^ T-cell count 300 cells μl^−1^ at month 1 increased significantly the risk of subsequent opportunist infection (Fernández-Ruiz M, 2013, unpublished data; Figure 2). Similarly, the depletion of the CD4^+^ T-cell subset is also useful to predict *de novo* post-transplant malignancy—another complication clearly related to over-immunosuppression—in the long term.^50, 51, 52^ The enumeration of peripheral blood lymphocyte sub-populations is technically simple, has a short turnaround time and may be performed in a fully automated way. In addition, the interpretation of its results appears easily intuitive to the clinician. However, we still need more studies to validate the prognostic accuracy of this strategy in different types of transplant recipients who have or have not received lymphocyte-depleting antibodies.

### Soluble CD30

CD30 is a 120 kDa transmembrane glycoprotein belonging to the tumor necrosis factor/nerve growth factor receptor superfamily.^53^ As well as being a classic marker for malignant cells of Hodgkin's lymphoma, CD30 has been recently implied in the regulation of the balance between Th_1_ and Th_2_ responses and the generation of T-cell memory.^54, 55^ A soluble form of CD30 (sCD30) of 85 kDa is cleaved in the bloodstream from the surface of activated T cells^56^ and its serum concentrations may be used as a functional marker for T-cell responsiveness.

In a seminal study based on a multicenter cohort of kidney transplant recipients, the long-term graft survival was significantly diminished in those with high pre-transplant serum levels of sCD30, with most of graft losses because of acute rejection.^57^ The kinetics during the post-transplant period have been also found to be useful in predicting alloreactivity, as recipients with a delayed decrease in sCD30 levels within the first week have a higher incidence of rejection.^58^ These findings have been externally validated by other investigators mainly in the kidney transplant setting,^59, 60, 61, 62, 63^ with somewhat discordant results for other types of transplants.^64, 65, 66^ Nonetheless, a recent meta-analysis concluded that the pre-transplant levels of sCD30 exhibit only modest sensitivity and specificity values (0.70 and 0.48, respectively) for the subsequent occurrence of acute graft rejection,^67^ suggesting that the serial monitoring of this biomarker throughout the post-transplant follow-up may be more reliable for this purpose.

The above evidence raises the question whether the measurement of sCD30 levels may have a role in predicting infection in SOT recipients. Unfortunately, only a few single-center studies have explored this approach.^61, 62, 64, 68^ Nikaein *et al.*^64^ reported that heart transplant recipients with pre-transplant sCD30 serum levels <90 IU ml^−1^ had a higher 1-year cumulative incidence of infection compared with those above this cutoff value, although this finding was not tested by multivariate analysis. Surprisingly, the same group found that high pre-transplant levels were associated with the occurrence of infection after kidney transplantation, failing to provide a plausible explanation for these opposite results.^68^ In a large cohort of kidney transplant recipients, Wang *et al.*^61^ reported that pre-transplant sCD30 serum levels <120 U ml^−1^ were predictive for the development of post-transplant pneumonia after adjusting by other clinical variables, and suggested as underlying pathogenic mechanism that the low expression of CD30 by T cells could result in decreased production of interleukin-13, which in turn has a role in recruiting inflammatory cells into the lung. In another study, the same authors measured sCD30 concentrations at regular intervals until month 60, and found that recipients with pneumonia had significantly lower levels during the first 3 months than those without.^62^ From a technical point of view, monitoring of sCD30 has various potential advantages, including its molecular resistance to repeated thawing cycles, the availability of a commercial enzyme-linked immunosorbent assay with good intra- and inter-assay reproducibility, and the low volume of serum required (25 μl).^69^ Nevertheless, the real accuracy of this approach, as well as the optimal cutoff values, have still to be validated in separate cohorts and different types of infection before being implemented in clinical practice.

### Intracellular concentration of ATP in stimulated CD4^+^ T cells

To date, the *in vitro* measurement of intracellular ATP (iATP) levels in peripheral blood CD4^+^ T cells following nonspecific stimulation with phytohemagglutinin is one of the few well-established strategies for functional immune monitoring in SOT recipients.^70^ The existence of a commercial assay (ImmuKnow; Cylex Inc., Columbia, MD, USA) approved by the Food and Drug Administration (FDA) in 2002 has contributed to this circumstance,^71^ as well as the amount of literature devoted since then to determine its real value for predicting post-transplant complications.^72, 73, 74^

Exposure of T cells to a mitogenic stimulus, such as phytohemagglutinin, leads to their metabolic activation and polyclonal expansion, a process in which the ATP synthesis and release precedes surface receptor expression, cytokine production and other subsequent events.^7^ Thus, the increases in iATP levels offer a proxy for the degree of functionality of the cell-mediated immune response.^75^ The protocol of the ImmuKnow assay is relatively simple. Heparinized whole blood is incubated with or without phytohemagglutinin (negative control) at 37 °C and 5% CO_2_ for 15–18 h. Paramagnetic particles coated with a monoclonal antibody to the human CD4 epitope are used to select CD4^+^ T cells from both the stimulated and non-stimulated wells. A lysing reagent is then added to release the iATP, which is measured by a luciferin/luciferase chemiluminescence method and expressed in ng ml^−1^.^76^ A population-based study comparing the assay results in healthy controls and SOT recipients established three categories to define patient's cell-mediated immune response: strong (⩾525 ng ml^−1^), moderate (226–524 ng ml^−1^) and low (⩽225 ng ml^−1^).^76^ Interestingly, iATP levels show a poor correlation with calcineurin inhibitor trough levels, total lymphocyte count or Th_1_/Th_2_ ratio.^76, 77, 78, 79^ Numerous authors have analyzed the predictive value of iATP for acute rejection, as recently summarized in a meta-analysis that found a relatively high specificity (0.75) but a low sensitivity (0.43), with significant heterogeneity across studies.^73^ The occurrence of post-transplant infection was also included as a primary outcome in most of these studies, encompassing kidney,^80, 81, 82^ liver,^79, 83^ heart^77, 84^ and lung^78, 85^ transplant populations. The frequency of monitoring and duration of follow-up differed, as well as the cutoff values used for iATP levels. Of note, the majority of studies analyzed post-transplant infection as an overall outcome, regardless of the nature of the complication or its pathogen. In the aforementioned meta-analysis, the pooled estimates for the predictive accuracy of iATP levels were poor (sensitivity of 0.58 and specificity of 0.69).^73^ A second meta-analysis focused only on liver transplant recipients reported better performance values (0.83 and 0.75, respectively).^74^ The utility of this approach has been also assessed for predicting some specific entities—including CMV and EBV infection,^86^ polyomavirus BK-associated nephropathy^81^ or invasive fungal infection^79^—usually in single-center studies with small sample sizes. A further application of the ImmuKnow assay could be predicting the progression from fungal colonization to invasive infection.^85^ A limitation shared by numerous studies lies in the indication for testing, triggered by the clinical suspicion of complication (that is, fever or deterioration of graft function) instead of being performed within a scheduled strategy for monitoring. The attribution of causality can be biased in the former setting, as the pathogen itself may be responsible for lowering iATP levels through mechanisms of T-cell exhaustion.^87^ On the other hand, the relative contribution of T-cell responsiveness (as measured by iATP concentrations) in controlling the infectious process differs according to the nature of the pathogen and it is likely that, by analyzing post-transplant infection as a single overall outcome, the actual predictive value of the assay may be underestimated. Accordingly, Husain *et al.*^85^ found that the levels of iATP were lower in lung transplant recipients with CMV disease and other viral infection as compared with those with bacterial or fungal infections, primarily controlled by the innate immunity. In addition, some studies have failed to show an association between single time point iATP values and the development of infection in the subsequent 90 days, raising the question whether the detection of changes over time by serial monitoring could be a more reliable approach.^82^ Finally, the blood sample storage time between collection and testing has been recently demonstrated to act as an unexpected source of variability in the assay results.^88^

In conclusion, although a functional monitoring approach based on the determination of iATP in stimulated CD4^+^ T cells seems appealing for a number of reasons, the optimal application of this assay in clinical practice still remains to be determined.

### Summary

Although non-pathogen-specific assays show promise as an objective measure of immune function in SOT recipients, it remains to be seen how they would be utilized in clinical practice. Larger interventional trials that show their utility for adjustment of immunosuppression or modification of prophylaxis are needed. In addition, the timing and frequency of performance of these assays needs to be established. Further, a combination of nonspecific assays may have improved predictive value for infection or rejection compared with a single assay. Another non-pathogen-specific immune assay that combines the assessment of both innate and adaptive immune responses for transplant recipients is currently under development (personal communication, Cellestis Ltd, Melbourne, Australia).

## Pathogen-specific monitoring

### CMV-specific immune response

Human CMV is one of the major causes of infection-related morbidity in SOT recipients and entails a non-negligible mortality in the absence of specific prevention. In addition, CMV exerts an indirect detrimental impact on both patient and graft outcome through its immunomodulatory effects. Those recipients with no pre-existing CMV-specific immunity at the time of primary infection (that is, the seronegative recipient of an organ from a seropositive donor (D^+^/R^−^)) have the highest incidence of CMV disease.^89^ As other herpesviruses, following primary infection CMV enters into a state of lifelong latency within numerous cellular types, including macrophages, neutrophils, fibroblasts, endothelial and epithelial cells.^90^ The virus uses a large repertoire of immune-evasion mechanisms, such as the inhibition of human leukocyte antigen (HLA)-restricted antigen presentation.^91^ Those recipients that are seropositive for CMV at transplantation (R^+^) face the risk of either viral reactivation or superinfection (reinfection), which in turn may involve a wide range of clinical presentations, from asymptomatic viremia to life-threatening tissue-invasive infection.^89^ This risk is modulated by different variables (type of transplant, use of T-cell-depleting antibodies as induction therapy, maintenance immunosuppression regimen, co-infection with other herpesviruses or presence of certain polymorphisms in genes regulating innate immunity, among others).^92^

Owing to its extraordinary immunogenicity, CMV is able to trigger robust responses from virtually every arm of the immune system.^28^ However, the T-cell-mediated adaptive immune response is by far predominant in the protection against CMV. As recently reviewed,^93^ IFN-γ-producing CMV-specific CD8^+^ T cells have a crucial role in limiting CMV viremia during the initial acute phase of primary infection, whereas the CD4^+^ T-cell subset seems to be more relevant in establishing long-term immune control. Although cytotoxic CD8^+^ T cells may target a great variety of viral proteins (approximately 70% of the viral proteome), the responses against tegument phosphoprotein pp65 and immediate early-1 (IE-1) antigens are largely essential.^94^ Of note, CMV infection induces the accumulation of late-stage differentiated CD4^+^ and CD8^+^ T cells, which exhibit an immunophenotype of replicative senescence with loss of CD27 and CD28 expression.^95, 96^ Natural killer and regulatory T cells also contribute to these processes.^97, 98^ The enumeration and *ex vivo* assessment of the functionality of CMV-specific T cells is being increasingly advocated to categorize the actual risk of developing CMV disease in a given patient.^99^ In fact, the recent International Consensus on CMV suggests that these assays may be applicable in the clinical setting.^100^ Such an approach should allow to individualize the prophylaxis strategy against CMV: patients at low risk—with an adequate CMV-specific cell-mediated immunity—would no longer benefit from viral load surveillance and/or antiviral prophylaxis, whereas these efforts could be devoted to those patients unable to mount (in case of primary infection) or reconstitute (if reactivation) a proper response.^93^ Hence, the currently available assays for the monitoring of CMV-specific T-cell-mediated immunity are reviewed (Table 2), as well as the clinical evidence supporting their application.

#### QuantiFERON-CMV assay

The QuantiFERON-CMV assay (Cellestis Ltd) is a commercially available enzyme-linked immunosorbent-based assay that measures the release of IFN-γ mostly by CMV-specific CD8^+^ T cells after *in vitro* stimulation of whole blood with a pool of 22 immunogenic viral peptides. Most of them are mapped within pp65 and IE-1 proteins, and their presentation appears restricted by some widespread HLA class I haplotypes that encompass the most common HLA types present in the general population.^101, 102^ The QuantiFERON-CMV assay is technically simple to perform. A heparinized whole blood sample is drawn into three different tubes: one coated with the viral epitopes (CMV tube), one containing no antigen used as negative control (nil tube), and one containing the phytohemagglutinin or positive control (mitogen tube). After vigorous shaking, the tubes are incubated overnight at 37 °C. The supernatants are subsequently harvested and the levels of IFN-γ measured by enzyme-linked immunosorbent assay. According to the manufacturer, the results should be interpreted as follow: nonreactive at <0.2 IU ml^−1^ (CMV minus nil) and ⩾0.5 IU ml^−1^ (mitogen minus nil); reactive at ⩾0.2 IU ml^−1^ (CMV minus nil) and any value in the mitogen tube; and indeterminate at <0.2 IU ml^−1^ (CMV minus nil) and <0.5 IU ml^−1^ (mitogen minus nil).^101^ In some studies, non-reactive and indeterminate results have been analyzed together for statistical purposes. An alternative cutoff value at 0.1 IU ml^−1^ has been shown to perform better in some studies.^99, 103^ Although not FDA-approved, the QuantiFERON-CMV assay is *Conformité Européenne* marked for commercial use in Europe.

Most of the experience recently gained in CMV-specific immune monitoring in SOT recipients derives from the QuantiFERON-CMV assay (Table 3). Its prognostic value has been assessed for predicting CMV-related outcomes in different clinical scenarios:

- *Pre-transplant risk-stratification in seropositive patients.* The baseline assessment of the risk of CMV infection conventionally relies on the pre-transplant IgG serostatus, under the assumption that CMV-seropositive patients (R^+^) have pre-existing immunity. Nevertheless, Cantisán *et al.*^104^ have recently found that about one-third of R^+^ transplant candidates actually lack a proper CMV-specific cell-mediated response as assessed by QuantiFERON-CMV assay. Interestingly, these patients were more likely to develop post-transplant CMV replication than those with a reactive assay before transplantation. The authors concluded that this strategy may eventually contribute to reclassify the current risk-stratification, as R^+^ patients with a pre-transplant non-reactive assay should be managed as high-risk patients. A planned intervention study will test this hypothesis.
- *Late-onset CMV disease after discontinuing antiviral prophylaxis.* A single-center study recruited 108 patients at high risk for CMV disease not only on the basis of their D/R serostatus, but also because of the previous administration of antithymocyte globulin or the type of transplant. The QuantiFERON-CMV was tested at baseline and then monthly during the first 100 days (that is, the usual duration of antiviral prophylaxis with ganciclovir or valganciclovir). Late-onset CMV disease was less frequent in patients with a detectable IFN-γ response (⩾0.1 IU ml^−1^) at the end of prophylaxis as compared with those with a negative test (5.3% vs 22.9% *P*-value=0.038).^103^ These findings were recently validated in a larger, multicenter study only focused on D^+^/R^−^ patients, in which the test was performed after discontinuation of antiviral prophylaxis (median duration of 98 days) and 1 and 2 months later. Patients with a positive result at any time had a cumulative incidence of subsequent CMV disease at month 12 significantly lower than those with negative or indeterminate results (6.4%, 22.2% and 58.3%, respectively; *P*-value <0.001). The positive predictive value for protection from CMV disease of having a positive QuantiFERON-CMV result was 0.90, although the negative predictive value was only moderate (0.27).^105^
- *Spontaneous clearance of asymptomatic CMV viremia.* It is usually assumed that R^+^ patients are able to spontaneously control most episodes of asymptomatic, low-grade viremia resulting from CMV reactivation (or superinfection in D^+^/R^+^ patients) without the need for antiviral therapy. Lisboa *et al.*^106^ analyzed 37 recipients at intermediate risk for CMV disease (R^+^ patients not given T-cell-depleting antibodies or lung transplant recipients), and found that the occurrence of subsequent spontaneous viral clearance was higher in those with a reactive assay (⩾0.2 IU ml^−1^) at the onset of detectable viremia (92.3% vs 45.5% *P-*value=0.004). This result offers a preliminary evidence that the QuantiFERON-CMV assay may be useful in monitoring patients undergoing preemptive therapy and to guide the decision of initiating antiviral therapy according to the adequacy of their CMV-specific cell-mediated response.^99^

#### ELISpot assay

The enzyme-linked immunosorbent spot (ELISpot) assay detects the release of IFN-γ by CD4^+^ and CD8^+^ T cells in CMV antigen-stimulated peripheral blood mononuclear cells (PBMCs). The stimulating antigen may be obtained from individual CMV peptides (that is, pp65 or IE-1), peptide libraries or whole virus lysates. Unlike the QuantiFERON-CMV, which determines the concentration of IFN-γ per ml of whole blood, the ELISpot assay quantifies the production of IFN-γ by assessing the number of spot-forming units in a given number of PBMCs. The IFN-γ secreted by activated cells is locally captured by a monoclonal antibody coated on a microtiter plate, and subsequently detected by using a second horseradish peroxidase-labeled antibody. Finally, individual spot-forming units are quantified with an image analysis software.^28^ Most of the previous studies with this method have relied on in-house assays,^107, 108, 109^ although a standardized assay (T-Track CMV; Lophius Biosciences GmbH, Regensburg, Germany) has been recently commercialized and is *Conformité Européenne* approved, and other commercial assays are also under development (Oxford Immunotec Ltd, Abingdon, UK). Further disadvantages are the time-consuming nature of the technique as compared with the QuantiFERON assay, and the lack of standardized cutoff values for classifying the adequacy of the T-cell response.^99^ The clinical experience with the ELISpot is relatively limited. By performing a monthly monitoring throughout the first year after kidney transplantation, Abate *et al.*^107^ found that the occurrence of low-grade viremia preceded an increase in CMV-specific T-cell responses, and that D^+^/R^−^ patients on antiviral prophylaxis suffered from a delay in mounting CMV-specific immunity. Bestard *et al.*^108^ performed the ELISpot assay with different stimuli—pp65 and IE-1 peptides and whole virus lysate—in 137 kidney transplant recipients before transplantation. Patients who developed CMV infection had significantly lower pre-transplant anti-IE-1 T-cell responses compared with those who remained free of infection, with good sensitivity and negative predictive values (over 0.80 and 0.90, respectively) for cutoff values of 7–8 spot-forming unit × 10^3^ PBMCs. Interestingly, no differences were found in the T-cell responses against pp65 or virus lysate, thus suggesting that IE-1 is crucial for triggering an effective CMV-specific cell-mediated immunity. A recent study comparing the performance of the two IFN-γ release assays (QuantiFERON-CMV and ELISpot) reported a similar ability for predicting CMV infection, although the areas under receiver operating curves were only modest (0.66 and 0.62, respectively).^109^

#### Flow cytometric intracellular cytokine staining

The intracellular cytokine stating (ICS) is based on the detection by flow cytometry of diverse Th_1_ effector cytokines (usually IFN-γ or tumor necrosis factor-α) in PBMCs or whole blood after stimulation with viral peptides, whole virus lysate or CMV-infected immature dendritic cells.^99^ As well as the enumeration of the CMV-specific T cells, the ICS also allows their phenotypic characterization through cell surface markers, and therefore this approach is regarded as the ‘gold standard' for assessing the CMV-specific cell-mediated immunity. The ICS method has been demonstrated to be useful in predicting the occurrence of CMV disease after kidney,^110, 111, 112, 113^ heart^111^ and lung^111, 114, 115^ transplantation. In one of the largest studies to date, Gerna *et al.*^113^ used immature dendritic cells infected with an endotheliotropic strain of CMV (VR-1814) to stimulate PBMCs and found that the presence of ⩾0.4 CMV-specific CD4^+^ and CD8^+^ T cells μl^−1^ was protective against CMV disease. Interestingly, in the absence of CMV-specific CD4^+^ T cells, the CD8^+^ subset was not able to consistently control alone viral replication. Among the disadvantages of the ICS method should be noted are its labor-intensive character and the lack of standardization in stimulating antigen preparation or protective cutoff values, as well as the requirement for a flow cytometer.

#### MHC-tetramer staining

In brief, main histocompatibility complex (MHC)-tetramers are complexes formed between HLA class I or class II molecules and antigenic peptides covalently linked to a fluorochrome, in order to allow the direct visualization of antigen-specific receptor-carrying T cells by using flow cytometry.^116^ Such tetramers are typically constructed by refolding MHC molecules in the presence of high concentrations of the antigenic peptide, followed by biotinylation of one chain of the MHC molecule. The resulting MHC-peptide complexes are bound in a 4:1 ratio to fluorophore-labeled streptavidin, thanks to the high affinity of the streptavidin–biotin interaction. The MHC-tetramer technology posses various advantages, including its high specificity, as well as the opportunity for surface and intracellular phenotyping or combination with functional assays.^117^ On the other hand, as the method is both epitope specific and HLA specific, it requires the knowledge of the individual HLA-type of the patient and large panels of tetramers should be available to be routinely implemented.^28^ Most of the clinical experience in the monitoring of the CMV-specific immunity by using this technology derived from hematopoietic stem-cell transplant recipients, with few studies in the SOT setting.^116, 118, 119^ The recent commercialization of a *Conformité Européenne*-marked assay (Dextramer CMV Kit; Immudex ApS, Copenhagen, Denmark), which uses different haplotypes covering about 95% of the European population, may contribute to the dissemination of this technique.

#### Other strategies for monitoring of CMV-specific immunity

It has been proposed that monitoring the expression of inhibitory costimulatory molecules on the surface of CMV-specific T cells, as a phenotypic marker of impaired immunity, may be useful in stratifying the risk for CMV infection. The upregulation of CD279, also known as programmed death-1 receptor, on total and CMV-specific CD8^+^ T cells was significantly associated with the development of CMV disease in a small group of high-risk (D^+^/R^−^) liver transplant recipients after discontinuation of antiviral prophylaxis.^120^ The serum levels of interleukin-10 showed a correlation with the overexpression of programmed death-1.^121^ In accordance with these findings, our group found that the frequencies of regulatory T cells (CD25^+^ FoxP3^+^)—which exert their function through the release of interleukin-10 and other inhibitory cytokines—were significantly higher at the onset of CMV viremia in patients failing to control the infection, and that having a ratio of CMV-3-specific CD4^+^ T cells to regulatory T cells above 0.4 predicted spontaneous clearance with good sensitivity and specificity values.^98^ Recently, Dirks *et al.*^122^ have proposed a rapid, stimulation-independent monitoring strategy based on assessing, by flow cytometry, the expression of programmed death-1 on CD28^−^ CD27^−^ CD4^+^ T cells. As CMV-specific T cells dominate among lymphocyte populations showing an immunophenotype of replicative senescence, the enumeration of CD27^−^ CD28^−^ CD4^+^ T cells could be used as a surrogate for CMV-specific immune response.

### Other virus-specific immune responses

In parallel to the above attempts to characterize CMV-specific immunity, some efforts have been focused on other relevant viral agents for the SOT population, mainly EBV and BKPyV.^123^

EBV possesses the capacity to establish lifelong latency within the B-cell compartment following primary infection.^124^ The control of latent EBV infection in healthy individuals depends largely on the status of cell-mediated immune response.^125^ In SOT recipients, EBV-induced B-cell proliferation may be poorly controlled and lead to post-transplant lymphoproliferative disease.^126, 127, 128^ Therefore, the monitoring of EBV-DNA replication in peripheral blood is routinely used to identify those patients at risk of developing this complication, particularly in the EBV-seronegative pediatric population.^129^ Previous studies have demonstrated the feasibility of measuring the EBV-specific T-cell-mediated response by MHC-tetramer staining,^130^ ICS^131^ and ELISpot assay,^9, 132^ although with limited clinical application at this time. One potential role of these approaches would be to monitor the effect of newer therapeutic interventions in patients with uncontrolled EBV replication (that is, adoptive cellular immunotherapy with donor EBV-specific cytotoxic T cells after *in vitro* expansion).^132^

The human BKPyV is linked to the development of polyomavirus BK-associated nephropathy in 1–10% of kidney transplant recipients, an important cause of premature and irreversible graft loss.^133^ BKPyV is a small, non-enveloped, double-stranded DNA virus with a widespread distribution in the general population that establishes latency in urogenital epithelial cells.^134^ Failure to activate BKPyV-specific cell-mediated immunity in the setting of post-transplant immunosuppression leads to viral reactivation and, eventually, the occurrence of progressive cytopathic changes, interstitial fibrosis, and tubular atrophy in the renal graft.^135^ Some studies have analyzed, by means of IFN-γ ELISpot assay, the kinetics of the BKPyV-specific cellular immunity directed against various viral proteins (that is, nonstructural small and large T antigens and structural VP1-3 proteins).^10, 136^ Schachtner *et al.*^136^ found that the increase in the frequency of BKPyV-specific T cells was associated with the spontaneous clearance of viral reactivation and with the recovery of graft function after tapering of immunosuppression in kidney transplant recipients with biopsy-proven polyomavirus BK-associated nephropathy. Although still preliminary, this monitoring strategy could therefore be useful in guiding the optimal decrease of immunosuppression in presence of polyomavirus BK-associated nephropathy in an attempt to minimize the associated risk of graft rejection.

## Future directions

Although notable advances in the development of novel strategies for post-transplant immune monitoring have been achieved, the precise role of these approaches in daily clinical practice is still far to be defined. Most of existing studies with nonspecific strategies are limited by small sample sizes, heterogeneity in baseline risk profiles of patients and lack of precise assessment of the different infectious syndromes and causative agents. Such caveats apply particularly to functional methods such as the ImmuKnow assay, so future studies should be performed in well-characterized subgroups of SOT recipients and be ultimately aimed at establishing pathogen- and graft-specific cutoff values (that is, prediction of fungal infection in lung transplant recipients), instead of using generic population-based categories of risk. Immune response is not a static process, nor is the risk of infection throughout the months following transplantation,^2^ so dynamic assessments by means of repeated testing at different points (early, intermediate and late post-transplant periods) should be encouraged. The potential additive value of predictive scores combining different assays is also worth exploring. Regarding the specific immune monitoring for CMV, Table 3 summarizes the different clinical scenarios in which this approach has been explored to date, as well as some unmet needs in the management of CMV infection in SOT recipients. Similarly, further studies should ideally be carried out on specific risk groups (that is, patients previously treated with T-cell-depleting antibodies) and achieve an adequate sample size, likely requiring multicenter collaborations. In that sense, the standardization of technical procedures and protective cutoff values is critical. Hopefully, definitive evidence on the utility of post-transplant immune monitoring will emerge in the years to come from intervention clinical trials.

## Figures and Tables

**Table 1:** Summary of proposed methods for non-pathogen-specific immune monitoring in SOT recipients

*Characteristic*	*Serum immunoglobulins*	*Serum complement factors (C3, C4, MBL)*	*Peripheral blood lymphocyte sub-populations*	*Soluble CD30*	*iATP in CD4*^+^ *T cells (ImmuKnow assay)*
Required sample	Serum	Serum	Whole blood	Serum	Whole blood
Assay	Nephelometry	Nephelometry or ELISA	Flow cytometry	ELISA	Quantification of iATP release in PHA-stimulated CD4^+^ T cells
Functional analysis	No	No	No	Yes	Yes
Advantages	Economical and easy to perform. Potential for replacement therapy with IVIGs	Economical and easy to perform. Potential for genotyping of *mbl2* gene variants	Easy to perform (automatized methods)	Easy to perform. Commercial assay. Low volume of serum required (25 μl)	Only FDA-approved commercial assay. Highly standardized. Large volume of studies
Limitations	Lack of standardized cutoff values. No information on the functionality of the humoral response	Lack of standardized cutoff values. No information on the functionality of the complement system	Lack of standardized cutoff values. No information on the functionality of the cellular response	Only few studies on predicting infection with discordant findings	Only modest PPV and NPV in studies to date. Relatively high cost. Potentially biased by sample storage time

Abbreviations: ELISA, enzyme-linked immunosorbent assay; FDA, Food and Drug Administration; iATP, intracellular adenosine triphosphate; IVIGs, intravenous immunoglobulins; MBL, mannose-binding lectin; NPV, negative predictive value; PHA, phytohemagglutinin; PPV, positive predictive value; SOT, solid organ transplantation.

**Table 2:** Currently available methods for monitoring of CMV-specific T-cell-mediated immune response in SOT recipients (modified from Egli *et al.* ^93^)

*Characteristic*	*QuantiFERON-CMV*	*ELISpot*	*Intracellular cytokine staining*	*MHC-tetramer staining*
Required sample (volume)	Whole blood (3–5 ml)	PBMCs (10 ml)	PBMCs or whole blood (1–2 ml)	PBMCs (0.5–1 ml)
Turnaround time	24 h	24–48 h	8–10 h	1–2 h
Antigen	Pool of 22 different peptides mapped within pp65, pp50, IE-1, IE-2 and gB	Individual peptide/peptide library/whole virus lysate/CMV (VR-1814)-infected immature dendritic cells	Individual peptide/peptide library/whole virus lysate/CMV (VR-1814)-infected immature dendritic cells	Individual peptide (pp65, IE-1, pp50)
Functional analysis	Yes	Yes	Yes	No (unless associated to intracellular cytokine staining)
Phenotypic characterization	No	No	Yes	Yes
Differentiation between CD8^+^ and CD4^+^ T cells	No (detects mostly CD8^+^ T cells)	No	Yes	Yes
Required knowledge on epitope	No	No	No	Yes
Required knowledge on individual HLA-type	No	No	No	Yes
Advantages	Simple to perform and highly standardized. CE-approved commercial assay with increasing clinical experience	CE-approved assay recently commercialized. Potential for freeze PBMCs and ship to reference laboratory for testing	Gold standard. Most data available with this technique. Potential for freeze PBMCs and ship to reference laboratory for testing	CE-approved assay recently commercialized. High specificity. Short turnaround time
Limitations	Not differentiation between CD8^+^ and CD4^+^ T cells. Sensitive to lymphopenia (high rate of indeterminate results in patients treated with ATG). Limited to widespread HLA types	Lack of technical standardization. No defined cutoff values. Need for purified PBMCs and access to an ELISpot reader. No differentiation between CD8^+^ and CD4^+^ T cells. Not FDA approved	Labor intensive. Lack of technical standardization. Need for access to a flow cytometer	Labor intensive. Lack of technical standardization. Need for purified PBMCs and access to a flow cytometer. Not FDA approved

Abbreviations: ATG, antithymocyte globulin; CE, *Conformité Européenne*; CMV, cytomegalovirus; ELISpot, enzyme-linked immunosorbent spot assay; FDA, Food and Drug Administration; gB, glycoprotein B; HLA, human leukocyte antigen; MHC, major histocompatibility complex; PBMCs, peripheral blood mononuclear cells.

**Table 3:** Clinical scenarios in which monitoring of CMV-specific T-cell-mediated immune response has been evaluated, and suggestions for future studies

*Clinical scenario*	*Predicted event*	*Previous studies*	*Monitoring method*	*Proposed intervention*
High-risk patients (D^+^/R^−^, T-cell-depleting antibodies, lung transplantation) during antiviral prophylaxis	Late-onset disease^a^	Yes^103, 107, 108, 137^	QuantiFERON-CMV, ELISpot	Prolong antiviral prophylaxis or close monitoring for viremia if inadequate response
High-risk patients (D^+^/R^−^) after discontinuing antiviral prophylaxis	Late-onset disease^a^	Yes^105^	QuantiFERON-CMV	Prolong antiviral prophylaxis or close monitoring for viremia if inadequate response
High-risk patients (T-cell-depleting antibodies, lung or pancreas transplantation) after discontinuing antiviral prophylaxis	Late-onset disease^a^	No		Prolong antiviral prophylaxis or close monitoring for viremia if inadequate response
Pre-transplant assessment in intermediate-risk patients (R^+^ with no other factors)	Post-transplant viremia and/or disease	Yes^104, 108^	QuantiFERON-CMV, ELISpot	Initiate antiviral prophylaxis in patients with inadequate response
Intermediate-risk patients (R^+^) on preemptive therapy with no concurrent viremia	Subsequent viremia and/or disease	Yes^108, 109, 110, 112, 113, 138^	ICS, QuantiFERON-CMV, ELISpot, MHC-tetramer staining	Reduce the frequency and/or discontinue monitoring of viremia if adequate response
Intermediate-risk patients (R^+^) on preemptive therapy with asymptomatic viremia	Spontaneous clearance	Yes^106^	QuantiFERON-CMV	Withhold antiviral therapy if adequate response
Active CMV infection or disease during antiviral treatment	Response to antiviral treatment	No		Decrease immunosuppression and/or modify antivirals if inadequate response
Active CMV infection or disease after discontinuation of antiviral treatment	Post-treatment relapse	Yes^115^	ICS	Initiate secondary prophylaxis if inadequate response
Acute graft rejection treated with steroid boluses and/or T-cell-depleting antibodies	Disease following anti-rejection therapy	No		Initiate prophylaxis if inadequate response

Abbreviations: CMV, cytomegalovirus; ELISpot, enzyme-linked immunosorbent spot assay; ICS, Intracellular cytokine staining; MHC, major histocompatibility complex.

a Refers to the occurrence of CMV disease after discontinuing antiviral prophylaxis with ganciclovir or valganciclovir (usually administered for 100–200 days).

## Notes

DK and AH have a research grant from Roche. MF-R has no conflict of interest. DK has received consulting fees from Oxford Immunotec.
